# Cone beam computed tomographic analysis of the spatial limitation during mandibular arch distalization

**DOI:** 10.1186/s12880-020-00441-y

**Published:** 2020-04-15

**Authors:** Chia-Ling Chen, Chia-Hui Chen, Chin-Yun Pan, Hong-Po Chang, Ping-Ho Chen, Yu-Chuan Tseng

**Affiliations:** 1 School of Dentistry and Graduate Program of Dental Science, College of Dental Medicine, Kaohsiung Medical University, 100 Shih-Chuan 1st Road, Kaohsiung, 80708 Taiwan; 2 Department of Orthodontics, Faculty of Dentistry, Kaohsiung Medical University, Hospital, Kaohsiung, Taiwan; 3 Department of Medical Imaging and Radiological Science, Central Taiwan, University of Science and Technology, Taichung, Taiwan; 4 Department of Dentistry, Kaohsiung Municipal Hsiao-Kang Hospital, Kaohsiung, Taiwan

**Keywords:** Mandibular arch distalization, Cortical contact, Cone beam computed tomography

## Abstract

**Background:**

In the literature, attempts are seldom made to quantify spatial limitation during mandibular arch distalization. This study aimed to investigate the spatial limitations associated with cortical contact with the mandibular second molar during mandibular arch distalization.

**Methods:**

The study population included 67 individuals who had undergone cone beam computed tomography (CBCT) (34 male and 33 female; mean age: 23.9 ± 2.72 years). The total ridge width, alveolar housing width, and root width were measured to evaluate the buccolingual limit. The space distal to the molar root represented the mesiodistal limit. The influence of sex, right versus left side, root-contact condition, malocclusion category, and presence of wisdom teeth were evaluated.

**Results:**

The rate of cortical contact was 49.3% before any orthodontic movement. No significant differences were observed in the alveolar width according to sex (male vs female), side assessed (right vs left), wisdom teeth (present vs absent), or malocclusion category. The ridge width and the alveolar width were smaller in the contact group than in the non-contact group (*P* < 0.01). The group with wisdom teeth showed a larger available distalization distance, but a significant difference was observed only near the alveolar crest.

**Conclusions:**

Both ridge width and available distalization distance were limiting factors for mandibular teeth distalization. For cases in which whole-arch distalization is planned, CBCT is recommended before treatment, especially for non-extraction treatment. This approach ensures safe and predictable tooth movement.

## Background

In orthodontic treatment, dentists can increase the available space in the dental arch with tooth extraction, arch expansion, interproximal enamel reduction, and arch distalization. Devices such as pendulum appliances, Schwarz plate-type appliances, Wilson distalization arches, distal jets, and sliding jigs can be used for this purpose [1–3]. However, the unintended effects of incisor proclination and molar tipping may result. For example, in a study by Joseph and Butchart [4], the distalization distance of the maxillary molar was 5.1 mm, but the maxillary incisor protruded labially by 3.7 mm. Moreover, the aforementioned procedures may induce changes in tooth inclination and anchorage loss that require correction [5].

Recently, temporary anchorage devices (TADs) have been developed to capitalize on the retromolar space. Among non-extraction studies, the average distalization of the upper molar was 1.64–2.8 mm, and the distal movement of the lower molar was 2.92–3.5 mm [6–8]. The use of miniplates and miniscrews can help achieve molar distalization distances of 3–5 mm [9]. For clinical cases at the borderline between extraction and non-extraction, clinicians can take advantage of bilateral distalization distance, which is approximately 6–10 mm.

Despite the changes in the dental arch during these procedures, adverse arch expansion and buccal and distal molar tipping cannot be ignored; conditions such as pericoronitis, pressure necrosis, root resorption, periodontal tissue resorption, and root exposure should also be considered [10, 11]. When the root moves beyond the alveolar housing, dehiscence and fenestration occur. If this movement is left unchecked, the root will continue to move into the submandibular fossa or maxillary sinus, after which, resorption damage is inevitable [10].

Although panoramic radiographs and lateral cephalograms have been used to predict the distalization distance, the buccolingual width cannot be estimated using these two-dimensional (2D) approaches. The mandibular second molar contacts the internal oblique ridge when it is distalized along the posterior occlusal line. The external oblique ridge, which is observed as the anterior border of the ramus on lateral cephalogram, is located anterolateral to the internal oblique ridge. Thus, the external oblique ridge is responsible for underestimation in measurements of the distance for molar distalization. Cone beam computed tomography (CBCT) imaging is suitable for visualizing and quantifying bone-structure morphology and changes associated with treatments [12].

In the literature, attempts are seldom made to quantify spatial limitation during mandibular arch distalization. We aimed to investigate the spatial limitations associated with cortical contact with the mandibular second molar during mandibular arch distalization analyzed through CBCT.

## Methods

The study protocol was reviewed and approved by the Institutional Review Board of Kaohsiung Medical University Hospital, Taiwan. This was a retrospective study conducted initially using 153 samples collected from patients who visited the Department of Orthodontics between January 2009 and April 2014. We included samples obtained from adults (mean age: 23.9 ± 2.72 years) with clear CBCT images. The exclusion criteria were a history of radiotherapy, oral and maxillofacial trauma/fracture, implant treatment, large restorations or amalgams, orthodontic treatment, and orthognathic surgery. A total of 67 cases were eventually selected. The assessments were focused on the mandibular molars and retromolar areas.

**Fig. 1:**
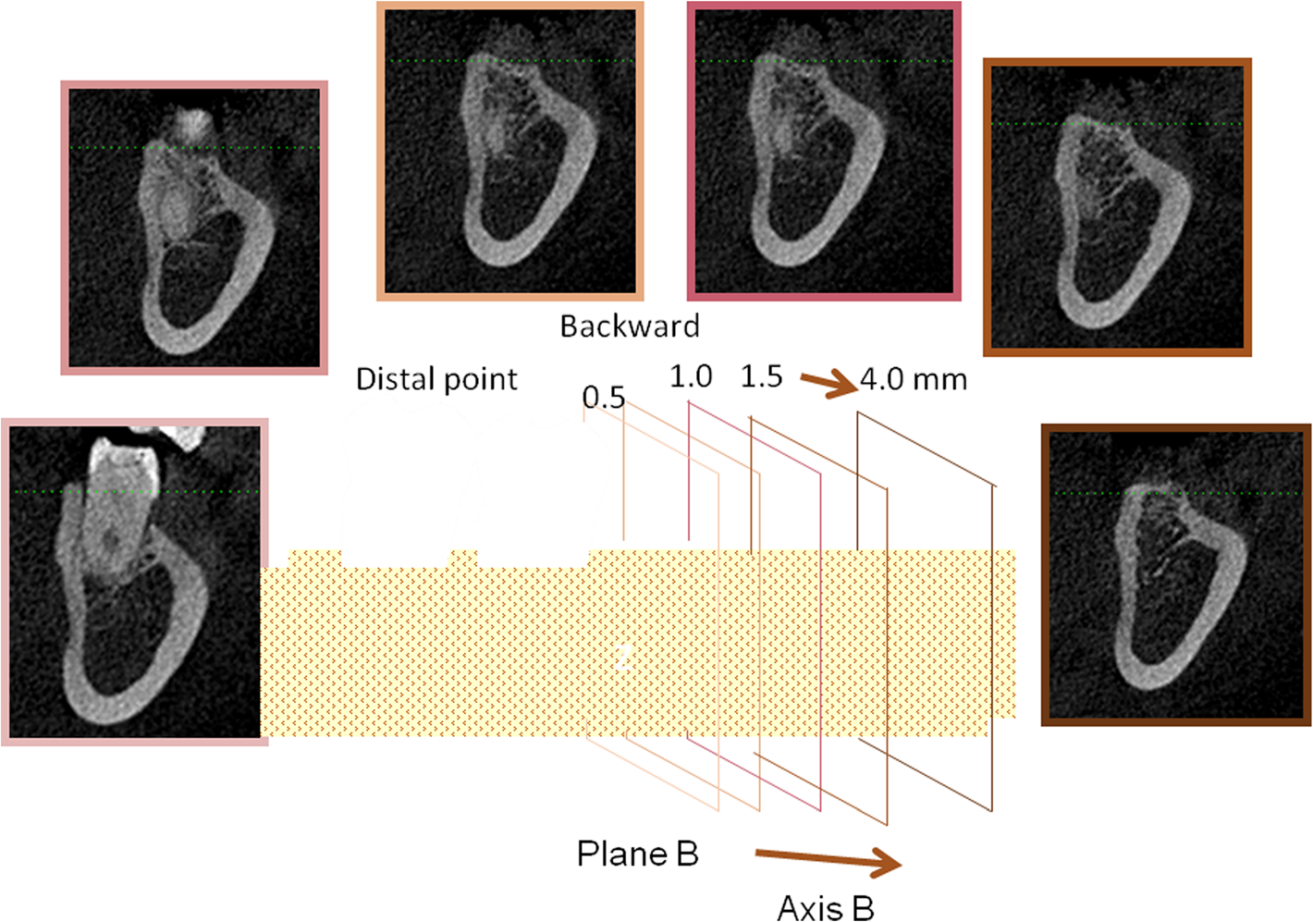
Plane B and its parallel planes

During the CBCT examinations (KaVo eXam; KaVo, Biberach, Germany), the participants maintained their heads in a natural position. The exposure settings were 26.9 s and 120 kV, the voxel size was 0.25 mm, and the field of view was 16 × 13 cm. Digital Imaging and Communications in Medicine (DICOM) data were measured using eXam Vision (KaVo eXam Vision; KaVo, Biberach, Germany). Plane A was defined as the occlusal plane and the Fiducial Line A was set as the occlusal line from the incisal edge of the mandibular incisor to the mesiobuccal cusp of the mandibular first molar. The right or left side was chosen randomly. The Fiducial Line B was a mesiodistal line that was equivalent to the average height of the alveolar crest of the mandibular first and second molars. The Fiducial Line C was the angle bisector of the axis of the mandibular first and second molars. Thus, even when the second molar tipped severely, the angle deviation would not be substantially affected. The Fiducial Line C was aligned with the direction of the apical movement in the axial view. Plane B was perpendicular to the Fiducial Line B and tangential to the most distal point of the lower second molar (Fig. 1). Measurements of the submandibular fossa and ridge width were manipulated in Plane B and moved along Line B.

**Fig. 2:**
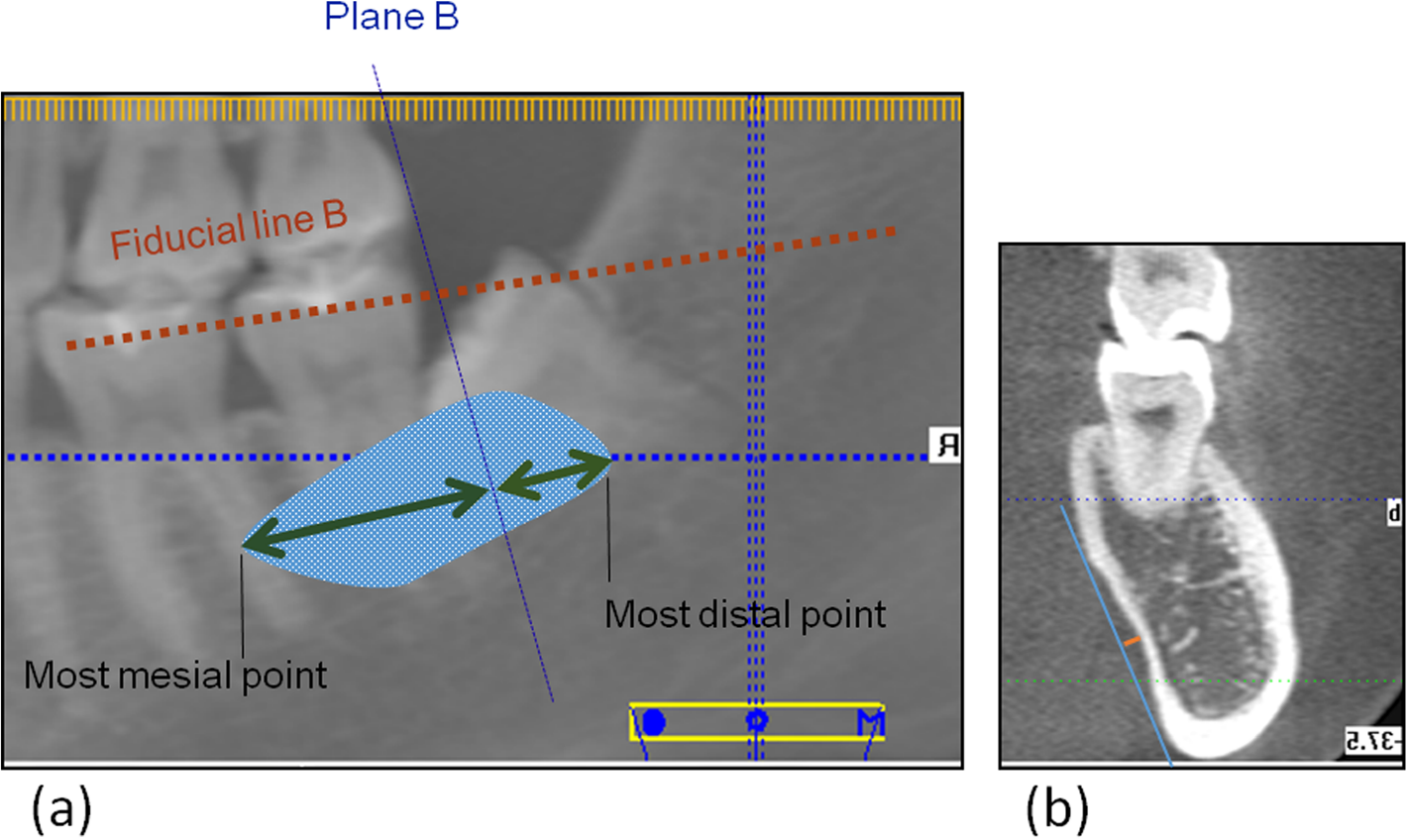
Measurement of submandibular fossa. (a) Submandibular fossa: blue line, plane B. (b) Section of plane B: orange line, fossa depth

In the first part of this study, we assessed the submandibular fossa. The submandibular fossa is a depression on the lingual surface of the body of the mandible inferior to the mylohyoid line. A tangent was drawn from the mylohyoid line to the lowest border of the mandible and the depth of the submandibular fossa was defined as the length from the tangent to the deepest point of the fossa (Fig. 2). The depth of definite submandibular fossa should be at least 1 mm and those with less than 1 mm were considered irregular anatomical structures. In addition, the boundary of the fossa was described relative to Plane B. The most superior and inferior points of the submandibular fossa were also determined to the average alveolar crest height of mandibular first and second molars. The length of the root was also measured from the average crest height to the root tip.

**Fig. 3:**
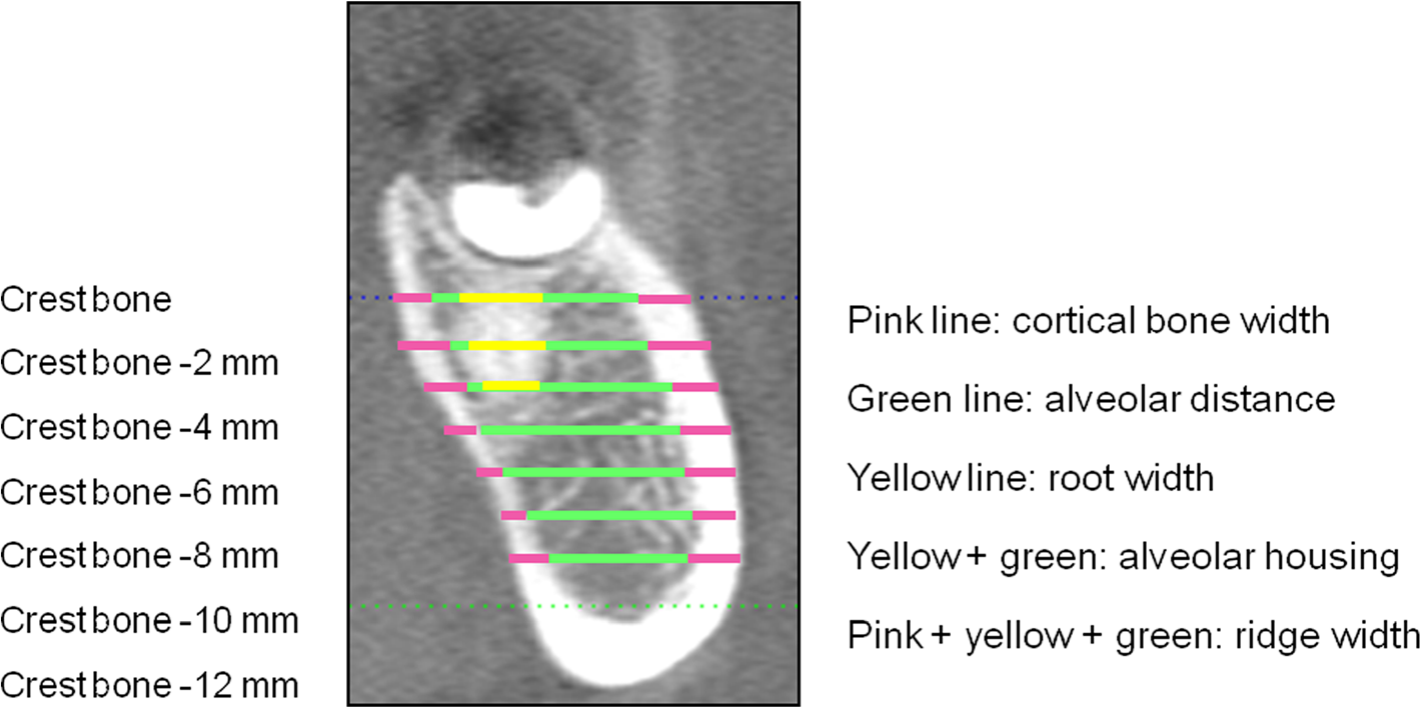
Measurement of total ridge width, alveolar housing and root width

The second part of this study was based on Plane B and its parallel planes. The first step involved measurement of the total ridge width, which contains the buccal and lingual cortex and the alveolar housing, as well as the root width. These measurements were taken at the level of the average alveolar crest of the mandibular first and second molars and at the subcrest 2, 4, 6, 8, 10, and 12-mm levels. The measurements were repeated on parallel planes with distalization of 0.5, 1.0, 1.5, 2, 2.5, 3, 3.5, and 4 mm (Fig. 3). These measurements were also taken on the plane containing the widest root. All these planes were perpendicular to the Fiducial Line B.

**Fig. 4:**
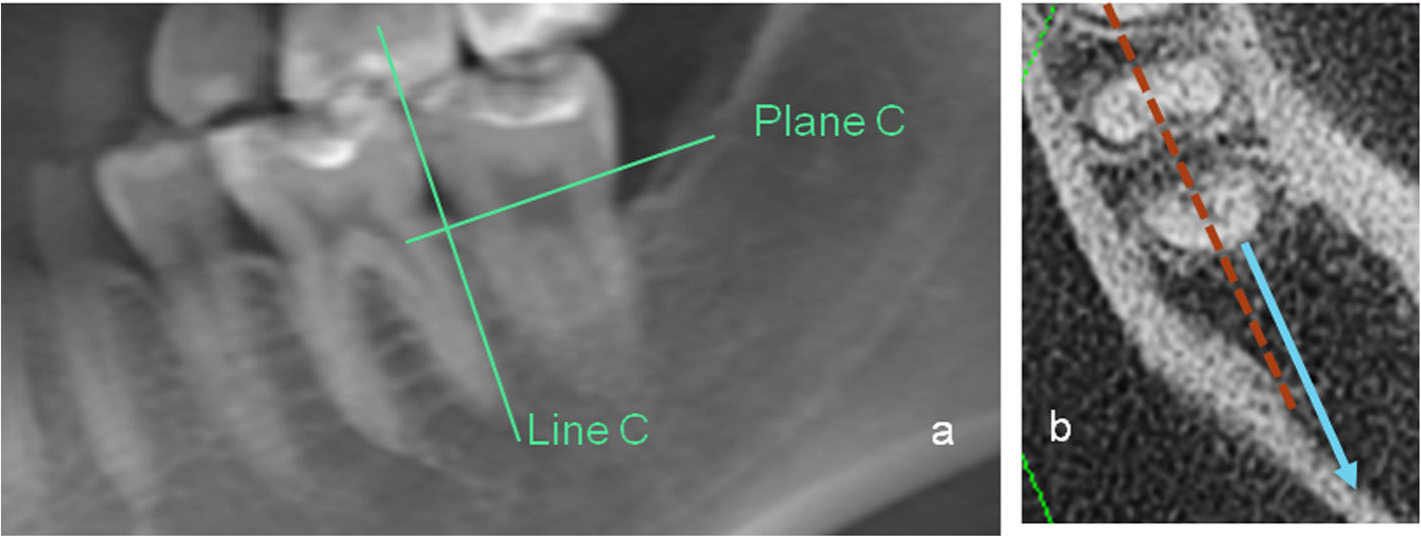
Available distalization distance. (a) Line C and Plane C. (b) parallel of Plane C, blue arrow: buccal distance; white arrow: lingual distance

The third part of this study evaluated the anatomical limit distal to the mandibular second molars (Fig. 4). Plane C was perpendicular to the Fiducial Line C and was at the level of the average crest height of the mandibular first and second molars. On Plane C, the available distalization distance was measured from the most distal part of the root to the nearest mandibular cortex. This distance was measured at the buccal and lingual sides. Likewise, the same process was performed on planes parallel to Plane C, with each plane 2 mm apical to Plane C. Along Line C, the measurements were performed at the subcrest 2, 4, 6, 8, 10, and 12-mm levels.

Statistical analysis was performed using SPSS for Windows (version 20; IBM, Armonk, NY, USA). The participants were classified by sex (male vs female), side assessed (left vs right), root-cortex contact, wisdom teeth (present vs absent), and malocclusion category. The chi-square test was used to compare the contact and non-contact condition, and the *t-*test was used to compare the ridge width and available distalization distance. The analysis of malocclusion category was performed using the Kruskal-Wallis test. Statistical significance was set at *p* < 0.01. All measurements were performed twice, by the same examiner, 2 weeks apart. The paired *t* test was conducted to verify the reproducibility of the measurements. The method error was examined using the Dahlberg formula [13]. The paired *t* test showed no statistically significant difference between the repeated measurements. The measurement errors for the total ridge width, alveolar housing width, and distalization distance were 0.12, 0.2, and 0.3 mm, respectively. The small difference in the measurements at the two observation times indicated reproducibility of the method. The rater was highly reliable between the repeated measurements.

## Results

The participants’ characteristics are presented in Table 1. The study population included 34 male and 33 female individuals. Left and right sides were assessed for 36 and for 31 participants, respectively. In 33 participants, wisdom teeth were found at the assessed side, whereas 34 participants did not have wisdom teeth on the assessed side. Malocclusion was categorized on the basis of Angle’s classification with molar relation. Class I, Class II, and Class III malocclusion were noted in 26, 16, and 25 participants, respectively. In 33 cases (49.3%), the roots of the mandibular molars were in contact with the cortical bone in the coronal and axial views. The chi-square analysis showed no significant differences in root-cortex contact according to sex (male vs female), side assessed (left vs right), wisdom teeth (present vs absent), or malocclusion category. When we evaluated the contact condition in the sagittal view, the mandibular molar was observed to be in contact with the internal oblique ridge with a contact ratio of 37.3% (25/67).

**Table 1:** Characteristics of the participants

	Root contact	Non-contact	*P* value*
Sex			
Male	17	17	1.00
Female	16	17	
Side			
Right	16	15	0.81
Left	17	19	
Wisdom tooth			
Yes	14	19	0.47
No	18	16	
Malocclusion			
I	13	13	0.51
II	6	10	
III	14	11	

*Chi-square test

We assessed the depth and position of the submandibular fossae and the root length of mandibular second molars (Table 2). The average depth of the submandibular fossa was 2.58 ± 0.82 mm. The most mesial point of the fossa was anterior to Plane B (tangential to the most distal point of the mandibular second molar) by 21.62 ± 6.41 mm. The most distal point of the fossa was posterior to Plane B by 12.92 ± 7.64 mm. The most superior and the most inferior points of the fossa were 7.87 ± 2.89 mm and 16.22 ± 2.51 mm beneath the alveolar crest, respectively. The average root length of the mandibular second molar was 11.09 ± 2.24 mm.

**Table 2:** Position and characteristics of the submandibular fossa and root length of the mandibular second molar

	Submandibular fossa					Root length
	Depth	Position				
		Most mesial point^a^	Most distal point^b^	Most superior point#	Most inferior point#	
Mean	2.58	21.62	12.92	7.87	16.22	11.09
SD	0.82	6.41	7.64	2.89	2.51	2.24

^a^anterior to Plane B (mm); ^b^posterior to Plane B (mm); #beneath alveolar crest (mm)

Plane B, perpendicular to the average height of alveolar crest of mandibular first and second molars and tangential to the most distal point of mandibular second molar

The measurements for the root width, alveolar housing, and total ridge width are presented in Table 3. No significant differences were observed in total ridge width according to the side (left vs right), wisdom teeth (present vs absent), or malocclusion category (*p* > 0.01). However, total ridge width showed significant differences between the sexes and between root-cortex contact and non-contact groups (*p* < 0.01). The total ridge width increased apically and distally. The total ridge width increased from the crest height down to the subcrest 4-mm level and then decreased apically. The width increased from the root area to the retromolar area but decreased from the plane that was 1 mm distal to Plane B.

**Table 3:** Width of the root, alveolar housing, and total ridge width (mm)

		Widest root area			Distal point of 2nd molar			0.5 mm distal to 2nd molar			1 mm distal to 2nd molar			1.5 mm distal to 2nd molar		
Subcrest		Root width	Alveolar housing	Ridge width	Root width	Alveolar housing	Ridge width	Root width	Alveolar housing	Ridge width	Root width	Alveolar housing	Ridge width	Root width	Alveolar housing	Ridge width
0 mm	Mean	8.07	9.03	12.05	6.38	8.01	12.96	5.56	8.03	13.07	5.40	8.27	13.52	0	8.22	13.59
	S.D.	2.91	3.31	4.53	3.32	4.25	5.15	5.24	4.99	5.40	3.39	4.22	5.03	0	4.54	5.29
2 mm	Mean	7.30	9.66	13.92	5.22	10.53	15.15	4.78	12.13	17.90	4.13	10.83	15.51	0	10.96	15.73
	S.D.	2.19	3.20	4.56	3.46	2.55	3.35	3.24	2.68	3.30	3.28	2.71	3.21	0	2.81	3.41
4 mm	Mean	6.54	11.20	16.42	5.18	10.95	17.02	4.36	10.94	17.22	4.00	11.07	17.30	4.65	11.25	17.54
	S.D.	1.90	3.09	4.60	3.23	2.46	2.69	3.13	2.21	2.40	3.12	2.46	2.61	2.85	2.14	2.28
6 mm	Mean	5.54	10.05	15.80	4.90	11.10	17.43	4.05	11.13	17.16	3.90	12.51	18.36	4.20	11.28	17.52
	S.D.	1.96	3.25	4.66	2.81	1.73	2.01	2.73	1.74	1.96	2.80	1.92	2.09	2.72	1.87	1.98
8 mm	Mean	4.78	10.09	15.82	4.26	10.78	17.13	3.77	10.76	15.36	3.70	10.71	16.91	3.58	10.88	15.90
	S.D.	2.42	3.07	4.45	2.52	1.73	1.89	2.29	2.10	2.28	2.40	2.10	2.19	2.25	1.92	2.07
10 mm	Mean	3.60	9.88	15.59	3.56	10.36	15.31	3.21	10.31	15.15	3.21	10.27	15.08	2.59	10.27	15.99
	S.D.	2.04	3.03	4.43	2.09	1.82	2.20	1.92	1.90	2.25	1.89	1.92	2.33	1.55	1.95	2.16
12 mm	Mean	2.96	9.19	14.93	3.22	9.55	15.28	2.45	9.73	15.12	2.77	10.55	15.04	2.28	9.58	14.91
	S.D.	1.12	3.28	4.71	1.35	2.39	2.97	0.88	2.26	2.84	1.02	2.28	2.87	0.81	2.28	2.80
		2.0 mm distal to 2nd molar			2.5 mm distal to 2nd molar			3.0 mm distal to 2nd molar			3.5 mm distal to 2nd molar			4.0 mm distal to 2nd molar		
Subcrest		Root width	Alveolar housing	Ridge width	Root width	Alveolar housing	Ridge width	Root width	Alveolar housing	Ridge width	Root width	Alveolar housing	Ridge width	Root width	Alveolar housing	Ridge width
0 mm	Mean	0	8.59	13.83	0	9.03	14.43	0	9.89	15.39	0	9.66	15.28	0	9.24	14.39
	S.D.	0	4.65	5.27	0	4.39	5.38	0	4.14	4.99	0	4.06	5.25	0	4.63	6.64
2 mm	Mean	0	10.91	16.72	0	11.17	17.11	0	11.12	17.00	0	10.98	16.84	0	10.34	15.74
	S.D.	0	2.83	3.38	0	2.65	3.15	0	2.97	3.69	0	3.27	4.17	0	4.31	5.98
4 mm	Mean	0	11.34	17.65	0	11.34	17.68	0	11.40	17.48	0	11.22	17.24	0	10.37	15.94
	S.D.	0	2.11	2.18	0	2.27	2.14	0	2.61	3.06	0	2.90	3.67	0	4.09	5.83
6 mm	Mean	5.30	11.42	17.68	0	11.13	17.42	0	11.12	17.28	0	10.85	16.90	0	9.98	15.61
	S.D.	2.64	1.90	1.97	0	1.98	2.18	0	2.36	2.94	0	2.74	3.60	0	3.92	5.73
8 mm	Mean	4.80	10.84	16.90	0	10.68	16.63	0	10.69	16.58	0	10.26	16.07	0	9.36	14.77
	S.D.	2.29	1.92	1.95	2.19	1.93	2.18	0	2.31	2.91	0	2.62	3.48	0	3.72	5.48
10 mm	Mean	2.83	10.04	15.79	2.90	9.97	15.72	2.00	9.80	15.46	0	9.46	15.12	0	8.66	13.86
	S.D.	1.30	2.28	2.45	1.53	1.85	2.23	1.23	2.16	2.87	0	2.44	3.45	0	3.49	5.22
12 mm	Mean	2.00	9.50	14.95	1.90	9.36	14.70	1.98	8.99	14.28	1.75	8.84	14.15	1.50	8.05	12.96
	S.D.	0.62	2.22	2.84	0.69	2.19	2.81	0.64	2.61	3.77	1.11	2.58	3.68	1.03	3.51	5.36

The alveolar housing was narrowest at the crest height, became wider apically, reached the maximum width at the subcrest 6-mm level, and decreased in width more apically (Table 3). When viewed from the second molars, the alveolar housing demonstrated an increase in width, with the maximum width noted on a plane 0.5 mm distal to Plane B. No significant differences were observed according to sex (male vs female), side assessed (left vs right), wisdom teeth (present vs absent), or malocclusion category (*p* > 0.01). The width of the alveolar housing in the root-cortex contact group was narrower than that in the non-contact group, and the differences were significant (*p* < 0.01).

The distances from the molar roots to the mandibular cortices are provided in Table 4, and these represent the available distalization distances for mandibular second molars. Most of the root contact was at the subcrest 10-mm level, with 13 such cases observed. The second most of the root contact was at the subcrest 8-mm level: 10 such cases were observed. Overall, the available distalization distance was larger in the buccal side than on the lingual side. In the non-contact group, the average available distalization space was 3.72 ± 1.69 mm, with the space reducing apically. From the crest to the apex (at 2-mm levels apically), the buccal spaces available for distalization were 5.96, 7.63, 7.38, 6.46, 4.98, 4.52, and 2.57 mm. The corresponding lingual spaces were 5.22, 5.84, 5.25, 3.96, 2.76, 2.27, and 1.81 mm. Sex, side assessed, and malocclusion category had no significant influence on the distalizing distance available (*p* > 0.01). However, the distalization distance available was larger in mandibles with wisdom teeth (*p* < 0.01). The results of this study showed that the lowest spatial limit was 1.81 mm for the whole-arch distalization.

**Table 4:** Available distalization space (mm)

Subcrest	Buccal side		Lingual side		*P*-value*
	Mean	SD	Mean	SD	
0 mm	5.96	5.46	5.22	4.98	0.024
2 mm	7.63	5.31	5.84	4.41	0.002
4 mm	7.38	5.10	5.25	4.51	0.001
6 mm	6.46	4.93	3.96	3.44	0.042
8 mm	4.98	4.71	2.76	3.36	0.001
10 mm	4.52	4.45	2.27	2.66	0.000
12 mm	2.57	3.93	1.81	2.71	0.002

*Independent *t* test

## Discussion

The introduction of TADs in orthodontic treatment enables predictable molar distalization with minimal patient compliance [7]. However, little is known about the posterior limit for the mandibular arch. Another issue that has received little attention to date is the limitation to the alveolar bone housing for posterior teeth cause by the inner and outer lingual cortices of the mandibular body. Currently, CBCT is the most complete and efficient imaging tool for diagnosis and planning of orthodontic treatment [14]. The purpose of this study was to investigate the spatial limitation associated with cortical contact with the mandibular second molar during mandibular arch distalization analyzed through CBCT.

Our analysis revealed that the roots of the mandibular second molar were in contact with the cortical bone in 33 out of 67 subjects (49.3%). In four cases, the contact point appeared in the axial view but not in the coronal view. In four other cases, the contact point appeared in the coronal view but not in the axial view. Thus, when contact was assessed only in a specific plane, the contact ratio was underestimated. Therefore, assessment should perform in at least two dimensions. When we evaluated the contact condition only in sagittal view, the contact ratio was 37.3% (25/67), which was similar to the contact ratio for cases of skeletal Class I with normodivergent facial profile as per a study (35.3%) [11]. In the serial studies conducted by Emes et al. and Aktop et al., the contact ratio of lingual soft tissue with the roots of mandibular third molars was 26–34.4% [15, 16].

The total ridge width, alveolar housing width, and available distalization distance demonstrated no significant difference according to the side of the mandible assessed or to malocclusion category. The total ridge width was wider in men than in women (*p* < 0.01), similar to the findings of Zhang et al. [17]. In the root-cortex contact group, the alveolar housing width was smaller at the subcrest 12-mm level, which was similar to the total ridge width. However, contact most frequently occurred at the subcrest 8- and 10-mm levels. Actually, the total ridge and alveolar housing were wide enough to contain the molar root, and the buccolingual width of the ridge only contributed partly to the contact. The most superior point of the submandibular fossa was 5.0–10.75 mm. Consequently, not only the size and position of the submandibular fossa but also the concavity of the fossa had an effect on the contact condition. Moreover, the distal limit and the position of the molar root must both be taken into consideration.

Prior to any tooth movement, the contact ratio was 49.3%. In the non-contact group, the least average space available for safe distalization was 1.81 mm. To safely distalize the whole arch, we recommend CBCT-based space assessments in advance of treatment planning. In the group for which wisdom teeth were present, the space for distalization was larger (Table 5), with a significant difference at the subcrest 2- and 4-mm levels (*p* < 0.01). In the group with wisdom teeth, the lingual side spaces available for distalization were 7.51, 7.69, and 6.57 mm. However, in the group without wisdom teeth, the corresponding spaces were 2.93, 3.99, and 3.93 mm. Regarding the maximum effect in the distal space, dental practicians can distalize the entire arch immediately after extraction of the wisdom teeth, which has the additional advantage of the regional acceleratory phenomenon [18]. The available distalization distance was greater in cases with Class II malocclusion, although no significant differences were observed among the three types of malocclusion (Table 5). Cases with Class I and Class III malocclusion required molar distalization, but the available space was smaller (*p* > 0.01).

**Table 5:** Available distalization space (mm)

			Sex		Side		Contact		Wisdom teeth		Malocclusion		
			Male	Female	Right	Left	Contact	Non-contact	Yes	No	I	II	III
0 mm	Buccal	Mean	5.11	6.74	4.41	6.99	6.36	5.56	7.37	4.55	4.36	7.28	7.01
		S.D.	5.44	5.47	5.17	5.48	5.65	5.34	5.72	4.89	5.01	6.51	4.99
	Lingual	Mean	5.00	5.42	4.00	6.04	5.61	4.84	7.51	2.93	5.22	6.62	4.23
		S.D.	5.30	4.76	4.51	5.18	5.36	4.64	5.38	3.29	5.50	5.07	3.83
2 mm	Buccal	Mean	7.99	7.30	6.74	8.22	7.58	7.68	9.24	6.02	8.92	7.16	6.37
		S.D.	5.12	5.56	5.67	5.07	5.14	5.58	5.57	4.60	4.53	6.71	5.07
	Lingual	Mean	6.58	5.16	5.12	6.32	5.93	5.75	7.69	3.99	6.82	6.61	4.08
		S.D.	4.45	4.35	4.58	4.31	4.21	4.61	4.40	3.64	4.72	5.06	3.03
4 mm	Buccal	Mean	7.17	7.57	6.63	7.87	7.04	7.71	7.86	6.89	8.31	6.89	6.56
		S.D.	4.77	5.48	5.45	4.89	4.97	5.31	5.65	4.56	3.34	8.00	4.35
	Lingual	Mean	4.77	5.70	4.41	5.82	4.78	5.72	6.57	3.93	5.58	6.96	3.64
		S.D.	3.61	5.24	3.56	5.02	4.36	4.70	5.32	3.09	3.44	6.87	3.16
6 mm	Buccal	Mean	6.22	6.68	5.87	6.85	5.85	7.07	7.42	5.50	6.73	7.60	5.32
		S.D.	4.23	5.58	4.84	5.04	5.26	4.61	5.83	3.72	3.34	7.85	3.98
	Lingual	Mean	3.63	4.26	3.59	4.20	3.48	4.43	4.85	3.07	4.44	4.83	2.74
		S.D.	2.74	4.02	3.06	3.70	3.45	3.44	3.96	2.61	3.00	5.16	2.07
8 mm	Buccal	Mean	4.42	5.49	4.75	5.13	4.19	5.76	6.08	3.87	5.55	5.79	3.69
		S.D.	4.24	5.13	4.98	4.59	4.72	4.65	5.52	3.49	3.99	6.50	4.03
	Lingual	Mean	2.45	3.04	2.25	3.09	2.35	3.16	3.65	1.86	3.02	3.28	2.06
		S.D.	2.70	3.91	2.84	3.68	3.44	3.30	4.01	2.32	3.19	4.80	2.30
10 mm	Buccal	Mean	4.34	4.68	4.60	4.46	3.69	5.34	5.55	3.48	5.61	4.72	3.02
		S.D.	4.46	4.53	5.19	3.97	2.08	4.42	4.97	3.68	4.30	5.74	3.30
	Lingual	Mean	2.30	2.24	1.99	2.46	1.68	2.86	2.87	1.67	2.88	2.37	1.45
		S.D.	2.74	2.65	2.69	2.67	2.46	2.77	2.74	2.50	2.84	2.75	2.29
12 mm	Buccal	Mean	2.23	2.88	2.70	2.48	1.72	3.41	3.23	1.90	2.50	4.20	1.49
		S.D.	3.65	4.21	4.35	3.69	3.35	4.33	4.63	3.02	4.28	4.99	2.01
	Lingual	Mean	1.44	2.15	1.34	2.12	0.87	2.74	2.40	1.22	2.08	2.42	1.04
		S.D.	2.30	3.05	2.35	2.92	2.05	3.00	2.94	2.37	2.93	3.41	1.66

**Fig. 5:**
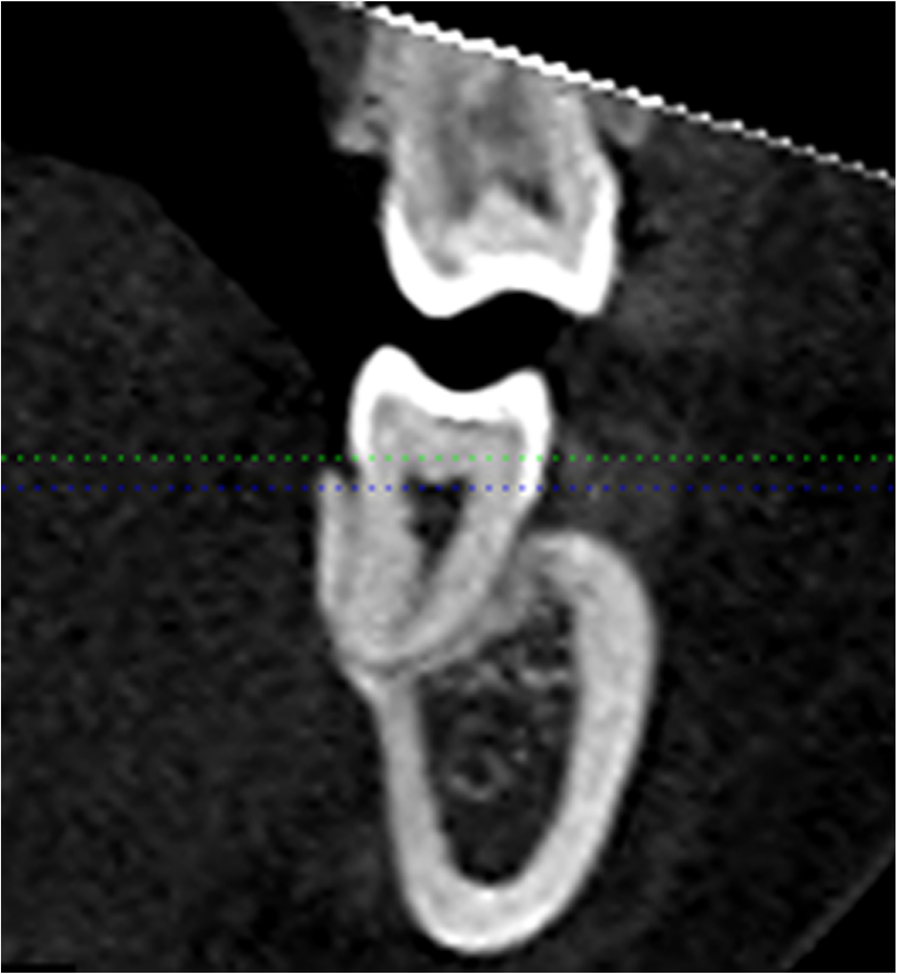
Contact condition: perforation

In cases where whole-arch distalization is planned instead of extraction, the patient should undergo root-cortex contact scanning first. For patients whose roots are in contact with the cortical bone, iatrogenic damage can occur if dental practicians distalize the molar unintentionally. For example, resorption of the root and periodontal tissue could occur when the root invades the cortical bone. In our study, the root had already invaded the cortical bone in two patients prior to any movement (2.99%), and the tip of the mandibular second molar root was found to be exposed in one of these cases (Fig. 5). As in other studies, dehiscence was also seen when the mandibular width was surveyed [19]. Therefore, before performing distalization, dental practicians should consider the available distance. If the most posterior mandibular teeth move too far, there may be no antagonist maxillary tooth.

In orthodontic treatment, thinner ridges tended to exhibit greater resorption [20]. Moreover, a thin ridge indicates a thin cortical bone [21]. Thin cortical bone is sensitive to forces and prone to microfracture, leading to vertical bony destruction. However, no changes occurred in alveolar bone ridge height at the maxillary lateral incisors with space opening in patients with congenitally missing maxillary lateral incisors [22]. Ridge preservation through orthodontic treatment could maintain the height at the lateral incisor regions [22]. Other factors can nevertheless influence these changes, for example, the direction of movement, the original position of the tooth, and the root-cortex distance [23]. The identification of more factors in future studies will provide dental practicians with a comprehensive overview of the interaction between tooth movement and alveolar height.

This study had some limitations. First, a priori power analysis was not used for the determination of the minimum required sample size. A power analysis should be considered during the design of the study to prevent drawing conclusions that are outside the study’s level of sensitivity. Statistical significance level was set at 1% and not at 5% in this study. A smaller *p* value as a means presents more significant findings. Second, soft tissue distal to the mandibular second molar was not taken into account. Clinically, there is thick soft tissue overlying the retromolar pad area that can result in considerably mandibular second molars being partially covered by the soft tissue. On the other hand, the lack of attached gingiva can be a limiting factor for molar distalization. An adequate amount of attached gingiva should be present around the retracted mandibular second molar to maintain periodontal health.

## Conclusion

The proportion of cases showing contact between the root and the cortex before orthodontic movement was 49.3%. Several factors were associated with root-cortex contact, including the ridge width, alveolar width, and distalization distance. Moreover, the size and position of the submandibular fossa, the position of the root, and the degree of contact on different planes influenced the final nature of the contact. In the non-contact group, the smallest available distalization distance was 1.81 mm. For cases in which whole-arch distalization is planned, CBCT is recommended before treatment, especially for non-extraction treatment. This approach ensures safe and predictable tooth movement.

## Data Availability

The datasets used and/or analysed during the current study are available from the corresponding author on reasonable request.

## Abbreviations

CBCT: Cone beam computed tomography
TADs: Temporary anchorage devices
